# Passive imaging with quantum advantage

**DOI:** 10.1126/sciadv.aei1896

**Published:** 2026-11-11

**Authors:** Li Gong, Aonan Zhang, Madhura Ghosh Dastidar, Alexander Duplinskii, A. I. Lvovsky

**Affiliations:** ^1^ Department of Physics, University of Oxford, Oxford OX1 3PU, UK.; ^2^ Quantum Innovation Centre (Q.InC), Agency for Science Technology and Research (A*STAR), Singapore, Singapore.

## Abstract

Far-field optical imaging inevitably involves low-pass spatial filtering, limiting the resolution. Moreover, conventional imaging suppresses high spatial frequency components close to the cutoff, making them invisible under noise—particularly the shot noise arising from discrete and random nature of quantum light. Here, we propose and implement a method for reducing the effect of this noise by optically preprocessing the incoming light before detection, thereby optimizing the quantum measurement performed on it. Our scheme, termed Fourier domain division (FDD), partitions the Fourier plane into multiple regions for independent detection and subsequent post-processing for image reconstruction. By analyzing the quantum and classical Fisher information, we show that our method is advantageous with respect to direct imaging for high spatial-frequency components. As a result, the number of photons required to achieve a certain signal-to-noise ratio in the Fourier domain is reduced, thus enhancing the overall resolution in the photon-starved regime. We demonstrate our method in microscopy, achieving fivefold improvement of Fisher information on high spatial frequency components. Unlike active super-resolution methods, FDD is passive, making it broadly applicable in microscopy and other imaging scenarios, where active illumination is impractical, including astronomy and remote sensing, in the photon-starved regime. Our work establishes a general strategy for designing quantum optimized superresolution imaging systems, bridging fundamental quantum limits, practical image analysis, and computer vision applications.

## INTRODUCTION

In conventional optical microscopy, spatial resolution is fundamentally constrained by diffraction, a direct consequence of the wave nature of light. Abbe interpreted this limit as a restriction in the Fourier domain of the image field imposed by the aperture of the objective lens (*1*). For incoherent intensity imaging, this results in the optical transfer function (OTF) of a nearly conical shape, meaning that the image is distorted in favor of low spatial frequency components, resulting in a further loss of resolution (*2*). To compensate for this loss, various deconvolution techniques are used. However, the capability of deconvolution rapidly degrades in the presence of noise (*3*), which can be both of fundamental and technical nature.

Here, we focus on the former case—namely, the shot noise, which results from the discrete and random nature of quantum light and becomes dominant under the conditions of low intensity. Motivated by the quantum estimation framework pioneered by Tsang *et al.* (*4*), we treat this issue as a quantum sensing problem. That is, we consider the quantum state of the optical field that passes through the objective lens and look for the measurement of this state that enhances the image reconstruction precision.

This approach enables us to use the tools from quantum estimation theory to quantify the measurement precision. Central to this framework is the notion of parameter—a quantitative feature of the quantum state that needs to be estimated. For a given set of parameters, the quantum Fisher information (QFI) sets the quantum Cramér-Rao bound (QCRB)—the lowest possible variance for an unbiased estimator permitted by quantum mechanics, minimized over all possible measurements (*5*). When the measurement is fixed, the achievable precision is governed by the classical Fisher information (FI) and its associated classical Cramér-Rao bound (CRB), which can never surpass the QCRB (*5*).

The traditional diffraction limit has been re-examined from this quantum perspective (*4*). A striking result has been that the QFI for estimating the separation between two incoherent point sources remains constant, independent of how small that separation is. The FI attainable with conventional direct imaging (DI) falls far below the QFI. On the other hand, the quantum limit can be saturated by measuring the optical field in the Hermite-Gaussian (HG) spatial mode basis (*4*). This strategy has been explored extensively in theory (*6*–*8*) and demonstrated in a series of experiments (*9*–*13*).

Imaging, however, is a much more complex task than point source separation. The complexity is due to not only a much larger parameter set but also correlations among the parameters and incompatibility between optimal measurements. HG basis measurement was proposed for imaging arbitrary two-dimensional objects (*14*) and shown to offer resolution advantage, albeit outside the quantum domain (*15*). Experimental validations now include coherent (*16*) and incoherent (*17*) imaging with wide-field illumination, as well as coherent imaging with a scanning, tightly focused beam (*18*). In the quantum domain, however, the advantage of HG imaging has not been established. More generally, imaging as a quantum sensing problem is not well-understood: The ultimate quantum bound for general imaging scenarios has not yet been analyzed nor even a functional parameter set for this task has been established.

This gap motivates our work on the quantum limit of imaging arbitrary samples and the design of optimal measurements. In our approach, we take the Fourier components of the sample intensity distribution as the parameters to be estimated, since accurately measuring high spatial frequency information is closely related to the resolution. We determine the QFI for this parametrization: Analytical calculations using reasonable approximations, validated by numerical simulations, provide insight into the design of optimized measurements. Accordingly, we propose a measurement scheme called Fourier domain division (FDD), in which the Fourier plane of the imaging system is divided into multiple regions, followed by intensity distribution measurement in the conjugate plane of the object. We develop an estimator to reconstruct the image from these measurements; our estimator is a generalized Wiener filter that effectively combines the information from all regions. Last, we demonstrate FDD experimentally using a fluorescent resolution target, achieving a 1.1-fold improvement in resolution and requiring fivefold fewer photons to reach the same estimation accuracy for high spatial frequency information.

The innovation of our work consists not only in developing a specific imaging method approaching the quantum limit. Converting the complicated quantum optimization task to a relatively simple OTF engineering problem establishes a general strategy for constructing quantum optimized imaging methods. This strategy can incorporate resolution enhancement tools such as structured illumination or two-photon fluorescence. By bridging quantum estimation theory and practical imaging, our approach establishes a pathway to fully exploit the quantum nature of light for super-resolution imaging of arbitrary samples.

## RESULTS

### Quantum estimation theory for imaging

An imaging system can be described as a low-pass filter in the Fourier domain, with the high-frequency components restricted by the objective lens aperture. The resolution is determined by the transmission and measurement precision of the high spatial frequency components.

To analyze the fundamental limits of an imaging system within the framework of quantum estimation theory, we first need a proper set of parameters that captures all resolvable structural information of the sample. Let the incoherent emission intensity distribution of the object (ground truth) be f(r). Its Fourier expansion is

$$f\left(r\right)=a_{0}+\sum_{\begin{array}{c} |k|\leq k_{c}\\ k_{x},k_{y}>0 \end{array}}a_{k}\text{cos}\left(k\cdot r\right)+b_{k}\text{sin}\left(k\cdot r\right)$$ (1)

We choose the amplitudes of the spatial Fourier components of the object $a_{k}$ and $b_{k}$ as the parameters to be estimated. f(r) is assumed normalized such that $\iint f\left(r\right)d^{2}r=1$. The spatial frequency k is truncated by the cutoff frequency $k_{c}=\frac{4\pi }{\lambda }\text{NA}$ of the incoherent imaging system under wide field illumination, where λ is the wavelength and NA is the numerical aperture (*2*). We assume the object to be of finite width so the Fourier series, rather than a continuous Fourier transformation, can be applied. This assumption simplifies the calculations but is not critical for the practical implementation of the method.

The quantum state of light in the image plane in each temporal mode is assumed to be in a weak thermal state (*4*, *19*), which can be modeled as $\hat{\rho }=\left(1-\delta \right)|0\rangle \langle 0|+\delta {\hat{\rho }}_{1}$, where δ is a small constant proportional to the emission rate and ${\hat{\rho }}_{1}$ is the one-photon state

$${\hat{\rho }}_{1}=\iint f\left(r\right)e^{-i\hat{K}\cdot r}|\Psi \rangle \langle \Psi |e^{i\hat{K}\cdot r}d^{2}r$$ (2)

In this equation, $|\Psi \rangle =\iint \Psi \left(r\right)|r\rangle d^{2}r$, Ψ(r) being the normalized amplitude point spread function of the imaging system (〈Ψ∣Ψ〉=1), ∣r〉 the photon position eigenket, and $\hat{K}$ the momentum operator, so that $e^{-i\hat{K}\cdot r}|\Psi \rangle$ is the quantum state of one photon under displacement r. Thus, ${\hat{\rho }}_{1}$ is the mixed state of a randomly displaced photon, with the classical probability distribution f(r). In this way, we convert the problem of “imaging an arbitrary sample” into the well-posed task of estimating the Fourier amplitudes from the quantum state of the collected photons.

For incoherent imaging, ${|\Psi \left(r\right)|}^{2}$ is the intensity point spread function (PSF) of DI. Its Fourier transform is the OTF $\beta^{\text{DI}}\left(k\right)=\iint {|\Psi \left(r\right)|}^{2}e^{-ik\cdot r}d^{2}r=\frac{1}{4\pi^{2}}\tilde{\Psi }\left(k\right)*\tilde{\Psi }\left(k\right)$, where $\tilde{\Psi }\left(k\right)$ is the Fourier transform of Ψ(r) and the asterisk denotes correlation. In typical microscopy, $\tilde{\Psi }\left(k\right)=\frac{4\sqrt{\pi }}{k_{c}}\theta \left(k_{c}/2-|k|\right)$ is a top-hat circular pupil function with cutoff frequency $\frac{2\pi }{\lambda }\text{NA}=\frac{k_{c}}{2}$, then $\Psi \left(r\right)=\frac{J_{1}\left(k_{c}r/2\right)}{\sqrt{\pi }r}$ is an Airy disk, where $J_{1}$ is the Bessel function of the first kind of order one. The QFI and FI of DI per detected photon can be calculated approximately as (see the derivation in section S1)

$${\text{QFI}}_{\mathrm{ij}}\approx \frac{\beta^{\text{DI}}\left(k_{i}\right)}{2a_{0}^{2}}\delta_{\mathrm{ij}}$$ (3)

and

$${\text{FI}}_{\mathrm{ij}}^{\text{DI}}\approx \frac{{\left[\beta^{\text{DI}}\left(k_{i}\right)\right]}^{2}}{2a_{0}^{2}\beta^{\text{DI}}\left(0\right)}\delta_{\mathrm{ij}}$$ (4)

where the parameters are represented as a single set $\left\{\theta_{i}\right\}=\left\{a_{k}\right\}\cup \left\{b_{k}\right\}$ and $k_{i}$ is the wavevector associated with $\theta_{i}$. Both are diagonal matrices, thus $\text{QCRB}={\text{QFI}}^{-1}$ and ${\text{CRB}}^{\text{DI}}={\left({\text{FI}}^{\text{DI}}\right)}^{-1},$ are also diagonal. The ratio between the FI of DI and the QFI is $\beta^{\text{DI}}\left(k\right)/\beta^{\text{DI}}\left(0\right)$ for a Fourier component with frequency k, tending to zero as ∣k∣ approaches the cutoff $k_{c}$. This suggests that DI is a substantially suboptimal measurement for estimating high frequency Fourier components of the object intensity distribution.

### Principle of FDD

FDD is an OTF engineering method aimed to maximize this ratio, thus enhancing FI. Figure 1A illustrates the principle of FDD in a standard 4-f imaging system. Light from the sample is Fourier-transformed by the first lens, truncated by a top-hat pupil function $\tilde{\Psi }\left(k\right)$ in the Fourier plane, and then imaged onto the detector by the second lens. In the Fourier plane, we insert a custom phase plate $\tilde{h}\left(k\right)$ that divides the support of the pupil function $\tilde{\Psi }\left(k\right)$ into several nonoverlapping regions associated with pupil functions ${\tilde{h}}_{l}\left(k\right)$. Each region is encoded with a distinct linear phase ramp $e^{-iR_{l}\cdot k}$, imparting a unique transverse momentum to the light passing through it. After the second lens, these phase-encoded regions give rise to multiple displaced images $g_{l}\left(r\right)$ in the image plane, each shifted by a displacement vector $R_{l}$. Then, the intensity distributions are measured with an array detector. The vector $R_{l}$ must be chosen to avoid overlapping between images from different pupils, so these images can be recorded simultaneously.

**Fig. 1.**
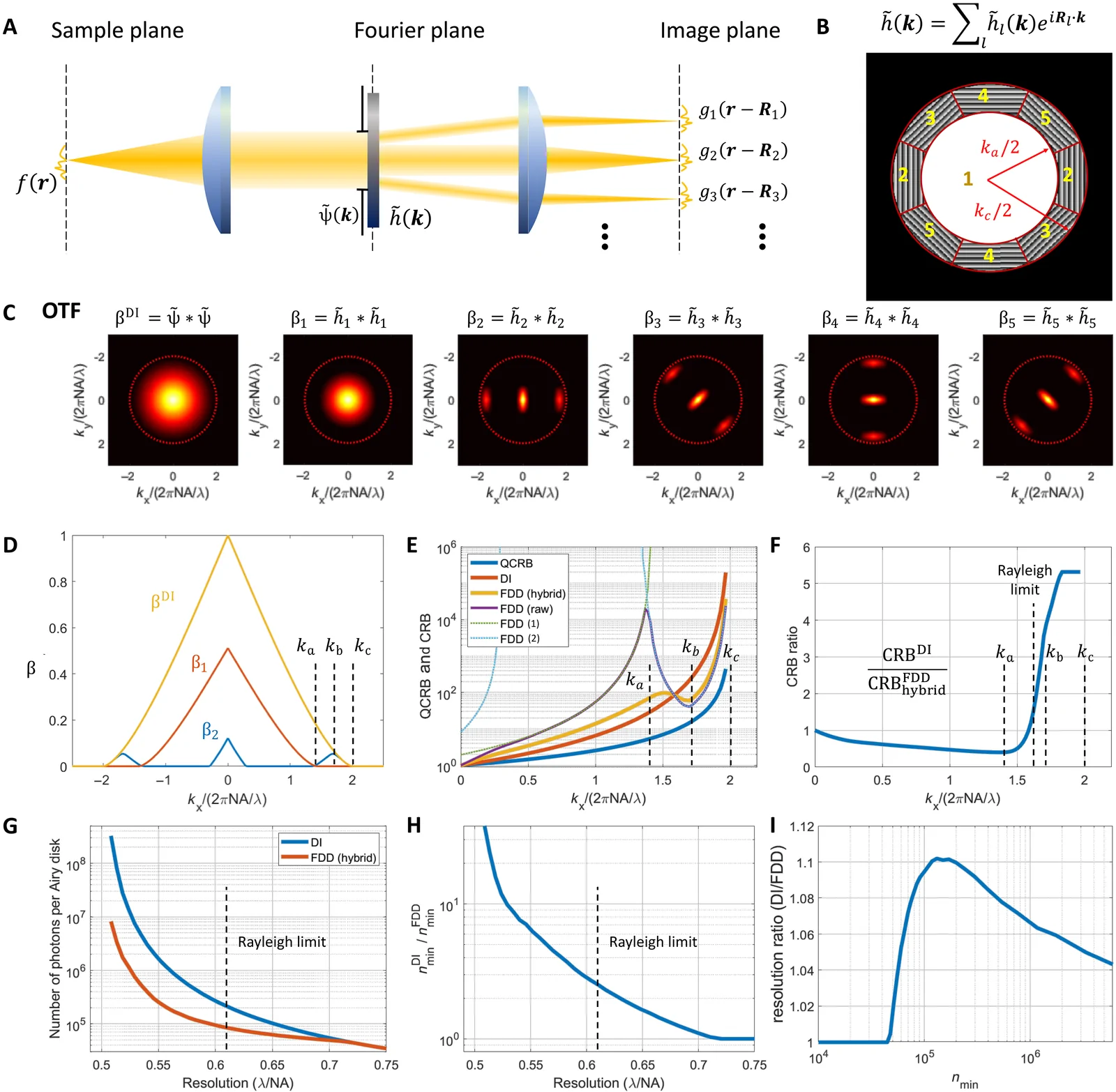
FDD concept. (**A**) Schematic of the FDD imaging system. A 4-f imaging system is modified by inserting a custom phase plate in the Fourier plane. (**B**) Example phase plate dividing the pupil into five regions ${\tilde{h}}_{l}\left(k\right),l=1,\ldots ,5$, each encoded with a different phase ramp to generate five separate images $g_{l}\left(r\right)$. (**C**) Corresponding OTFs as autocorrelation of the pupil. The red dashed circle indicates the cutoff frequency $k_{c}$. (**D**) Line profiles of the first and the second FDD OTFs, as well as the DI OTF, along the $k_{x}$ axis. (**E**) CRBs for DI, FDD (raw), FDD (hybrid), first and the second FDD channel [FDD(1) and FDD(2)], respectively, and the QCRB; α=0.6 is assumed. (**F**) CRB ratio between DI and FDD (hybrid). For high spatial frequencies, the CRB of FDD (hybrid) is about five times lower than that of DI, showing substantial advantage. (**G**) Minimum photon numbers $n_{\text{min}}^{\text{DI}}$ and $n_{\text{min}}^{\text{FDD}}$ per Airy disk area as a function of the achievable resolution. (**H**) Ratio between $n_{\text{min}}^{\text{DI}}$ and $n_{\text{min}}^{\text{FDD}}$. (**I**) Ratio of achievable resolutions between DI and FDD.

Figure 1B shows an example phase plate where the Fourier plane is partitioned into five regions ${\tilde{h}}_{l}$ (l=1,…,5), each encoded with a different linear phase ramp to generate five corresponding images. As labeled in Fig. 1B, the radii of $\tilde{\Psi }\left(k\right)$ and ${\tilde{h}}_{1}\left(k\right)$ are $k_{c}/2$ and $k_{a}/2$, respectively. The corresponding OTFs $\beta_{l}\left(k\right)=\frac{1}{4\pi^{2}}{\tilde{h}}_{l}\left(k\right)*{\tilde{h}}_{l}\left(k\right)$ are shown in Fig. 1C.

Figure 1D compares the $k_{x}$ axis cross sections of the OTF for DI ($\beta^{\text{DI}}$) with that of the first and the second pupil for FDD [$\beta_{1}\left(k\right)$ versus $\beta_{2}\left(k\right)$]. Since Ψ is normalized, $\beta^{\text{DI}}\left(0\right)=\langle \Psi |\Psi \rangle =1$, and each $\beta_{l}\left(0\right)$ is the area ratio between the FDD pupil function ${\tilde{h}}_{l}\left(k\right)$ and the DI pupil function $\tilde{\Psi }\left(k\right)$, e.g., $\beta_{2}\left(0\right)=\frac{\left(1-k_{a}^{2}/k_{c}^{2}\right)}{4}\ll 1$. On the other hand, $\beta^{\text{DI}}\left(k_{x}\right)\approx \beta_{2}\left(k_{x}\right)$ for high frequencies $k_{x}\geq k_{b}\equiv \frac{k_{a}+k_{c}}{2}$.

This behavior permits precise evaluation of the image Fourier components corresponding to these frequencies. To see this, we write the FI for the lth pupil of FDD (refer to section S1 for detailed calculation)

$${\text{FI}}_{\mathrm{ij}}^{\left(l\right)}\approx \frac{{\left[\beta_{l}\left(k_{i}\right)\right]}^{2}}{2a_{0}^{2}\beta_{l}\left(0\right)}\delta_{\mathrm{ij}}$$ (5)

Because the FI scales as ${\left[\beta_{l}\left(k_{i}\right)\right]}^{2}/\beta_{l}\left(0\right)$, the reduction of $\beta_{2}\left(0\right)$ boosts FI^(2)^ for high-frequency components by a factor of up to $\frac{4}{\left(1-k_{a}^{2}/k_{c}^{2}\right)}$, demonstrating a substantial advantage for estimating fine spatial detail. Intuitively, detecting the regions ${\tilde{h}}_{2}-{\tilde{h}}_{5}$ separately from the central region ${\tilde{h}}_{1}$ allows us to measure the contribution from high-frequency Fourier components without contamination from the shot noise associated with low frequencies, therefore enhancing the precision of these measurements.

The total FI of FDD is the sum of the FIs from each pupil

$${\text{FI}}_{\text{raw}}^{\text{FDD}}=\sum_{l=1}^{5}{\text{FI}}^{\left(l\right)}$$ (6)

As seen in Fig. 1E, ${\text{CRB}}_{\text{raw}}^{\text{FDD}}={\left({\text{FI}}_{\text{raw}}^{\text{FDD}}\right)}^{-1}$ shows substantial advantage with respect to ${\text{CRB}}^{\text{DI}}$ in the high-frequency region, with a minor loss in the low-frequency region. Unfortunately, however, it is singular for $k=k_{a}$. This is because the FDD OTF at this point vanishes (Fig. 1D). To address this issue, we redistribute the total photon budget, allocating a fraction α across the five segmented pupils, while the remaining 1−α are acquired via DI. This results in the final hybrid FI

$${\text{FI}}_{\text{hybrid}}^{\text{FDD}}={\mathrm{\alpha FI}}_{\text{raw}}^{\text{FDD}}+\left(1-\alpha \right){\text{FI}}^{\text{DI}}$$ (7)

As shown in Fig. 1E, this hybrid strategy ${\text{CRB}}_{\text{hybrid}}^{\text{FDD}}={\left({\text{FI}}_{\text{hybrid}}^{\text{FDD}}\right)}^{-1}$ suppresses the singularity while largely maintaining the enhancement for high k’s (Fig. 1F). As discussed above, the level and the frequency range of this enhancement are determined by the ratio $k_{a}:k_{c}$. Higher ratio leads to higher enhancement but narrower range. In our experiments, we choose $k_{a}=0.7k_{c}$, corresponding to $k_{b}=0.85k_{c}$, to cover the range of enhancement beyond the Rayleigh limit 0.61λ/NA, corresponding to $0.82k_{c}$. The photon splitting ratio α is chosen as 0.6, which provides close-to-optimal resolution enhancement for our measurement regime, as discussed below.

As evidenced by the behavior of the red curve in (Fig. 1E), the number of photons required to achieve a given precision in evaluating a particular Fourier component rapidly grows with frequency. Therefore, if our goal is to precisely evaluate a wider range of the image spectrum with a given photon budget, then it is beneficial to pick a measurement that sacrifices the FI for low frequencies in favor of high frequencies. This is exactly the vision of FDD.

To quantify this intuitive argument and find the achievable resolution for a given photon budget, we evaluate the minimum photon number $n_{\text{min}}$ per area of an Airy disk such that for all ∣k∣<k, the ratio between the true value of the parameter $\theta_{i}$ and its SD $\sqrt{<\Delta \theta_{i}^{2}>}$ is above a threshold γ. As we derive in section S2, this minimum photon budget for DI is

$$n_{\text{min}}^{\text{DI}}\left(k\right)\approx \frac{8\times 0.61^{2}\pi^{2}\gamma^{2}}{R^{2}}\underset{|k_{i}|<k}{\text{max}}\frac{\beta^{\text{DI}}\left(0\right)}{{\left[\beta^{\text{DI}}\left(k_{i}\right)\right]}^{2}}$$ (8a)

where $R=\frac{\sqrt{\langle \Delta f{\left(r\right)}^{2}\rangle }}{a_{0}}$, a real number typically below 1, is the coefficient of variation of the sample. For hybrid FDD, it is

$$n_{\text{min}}^{\text{FDD}}\left(k\right)\approx \frac{8\times 0.61^{2}\pi^{2}\gamma^{2}}{R^{2}}\underset{k_{a},\alpha }{\text{min}}\left[\underset{|k_{i}|<k}{\text{max}}{\left[\sum_{l=0}^{5}\frac{{\left[\beta_{l}^{H}\left(k_{i}\right)\right]}^{2}}{\beta_{l}^{H}\left(0\right)}\right]}^{-1}\right]$$ (8b)

where $\beta_{l}^{H}={\alpha \beta }_{l}$ for l=1,…,5, $\beta_{0}^{H}=\left(1-\alpha \right)\beta^{\text{DI}}$. Reversing these relations, the value of *k* can be calculated for both methods given the photon budget $n_{\text{min}}$. The achievable spatial resolution is then 2π/k.

Figure 1G plots $n_{\text{min}}^{\text{DI}}$ and $n_{\text{min}}^{\text{FDD}}$ as a function of achievable resolution 2π/k, assuming *R* = 0.4 and = 3. It shows that FDD requires a much lower photon budget in the high-resolution region. The ratio $n_{\text{min}}^{\text{DI}}/n_{\text{min}}^{\text{FDD}}$ is plotted in Fig. 1H; 2.5-fold improvement is found at the Rayleigh limit $\frac{2\pi }{k}=\frac{0.61\lambda }{\text{NA}}$, while the improvement approaches infinity for resolution close to $\frac{2\pi }{k}\to \frac{2\pi }{k_{c}}=\frac{0.5\lambda }{\text{NA}}$. Figure 1I plots the ratio of achievable resolution between DI and FDD versus the photon budget. FDD achieves up to 1.1-fold resolution improvement.

### Image reconstruction algorithm

We denote the total photon number as N, normalized ground truth intensity distribution f(r), and the recorded photon density (number of photons per area) for the lth pupil function as $g_{l}\left(r\right)=\langle g_{l}\left(r\right)\rangle +n_{l}\left(r\right)$, where $\langle g_{l}\left(r\right)\rangle$ is the mean value and $n_{l}\left(r\right)$ is the noise. In Fourier domain, ${\tilde{g}}_{l}\left(k\right)=\langle {\tilde{g}}_{l}\left(k\right)\rangle +{\tilde{n}}_{l}\left(k\right)$. The mean photon density is $\langle {\tilde{g}}_{l}\left(k\right)\rangle =N\beta_{l}^{H}\left(k\right)\tilde{f}\left(k\right)$, where $\tilde{f}\left(k\right)$ is the Fourier transform of the ground truth f(r). To reconstruct the image, the six raw images produced by FDD are combined in the Fourier domain using a generalized Wiener filter-type estimator (*20*) to yield the super-resolved reconstruction

$${\tilde{g}}^{\text{FDD}}\left(k\right)=\sum_{l=0}^{5}C_{l}\left(k\right){\tilde{g}}_{l}\left(k\right)$$ (9a)

with

$$C_{l}\left(k\right)=\frac{\beta_{l}^{H}\left(k\right)/\beta_{l}^{H}\left(0\right)}{\sum_{m=0}^{5}{\left[\beta_{m}^{H}\left(k\right)\right]}^{2}/\beta_{m}^{H}\left(0\right)+\epsilon_{\text{FDD}}\left(k\right)}$$ (9b)

where

$$\epsilon_{\text{FDD}}\left(k\right)=\frac{1}{N{|\tilde{f}\left(k\right)|}^{2}}$$ (9c)

is a small regularization term, which prevents division by zero. Because this term contains the ground truth information, the estimator is applied iteratively, first initializing $\epsilon_{\text{FDD}}\left(k\right)=N/\sum_{l}{|{\tilde{g}}_{l}\left(k\right)|}^{2}$ and, in subsequent iteration steps, applying $\epsilon_{\text{FDD}}\left(k\right)=\frac{N}{{|{\tilde{g}}^{\text{FDD}}\left(k\right)|}^{2}}$ from the output of the previous step. Last, the reconstructed image $g^{\text{FDD}}\left(r\right)$ can be obtained by inverse Fourier transform of ${\tilde{g}}^{\text{FDD}}\left(k\right)$. Full details are given in section S3, where we derive Eqs. 9a-c by minimizing the mean square error between ${\tilde{g}}^{\text{FDD}}\left(k\right)$ and the ground truth $N\tilde{f}\left(k\right)$. We prove that, in the limit $\epsilon_{\text{FDD}}\left(k\right)\to 0$, the estimator described in Eq. 9 attains the signal-to-noise ratio (SNR) allowed by the CRB.

For fair comparison with DI, we apply similar processing to the DI image, which in this case is equivalent to deconvolution by means of the conventional Wiener filter (*20*). Because DI has only a single pupil function $\tilde{\Psi }\left(k\right)$, Eq. 9 reduces to

$${\tilde{g}}^{\text{DI}\text{dcv}}\left(k\right)=C^{\text{DI}}\left(k\right){\tilde{g}}^{\text{DI}}\left(k\right)$$ (10a)

$$C^{\text{DI}}\left(k\right)=\frac{\beta^{\text{DI}}\left(k\right)}{{\left[\beta^{\text{DI}}\left(k\right)\right]}^{2}+\epsilon_{\text{DI}}\left(k\right)}$$ (10b)

$$\epsilon_{\text{DI}}\left(k\right)=\frac{1}{N{|\tilde{f}\left(k\right)|}^{2}}$$ (10c)

This estimator features a similar circular dependency, which can be solved by the same iteration algorithm, initializing with $\epsilon_{\text{DI}}\left(k\right)=N/{|{\tilde{g}}^{\text{DI}}\left(k\right)|}^{2}$.

### Experimental validation

The experimental setup is shown in Fig. 2. A continuous-wave laser at 473 nm (Toptica iBeam smart-473) illuminates a fluorescent resolution test chart (TC-RT03fl, Technologie Manufaktur GmbH & Co. KG), which consists of parallel line quintuplets with spatial frequencies labeled in lines per millimeter. The emitted fluorescence is collected by an oil-immersion objective lens (UPlanSApo 100×, NA 1.4, Olympus). The back aperture of the objective corresponds to the Fourier plane of the imaging system. A 10-nm bandpass filter (FBH540-10, Thorlabs) is used to isolate the fluorescence from the laser. A 4-f optical system, formed by lenses L1 ($f_{1}=500$ mm) and L2 ($f_{2}=300$ mm), images this Fourier plane onto a spatial light modulator (SLM, Meadowlark E19x12-500-1200-HDM8), where the pupil function $\tilde{h}\left(k\right)$ is displayed. Because of the limited range of phases that the SLM can apply at each location, the phase ramp is implemented as a blazed grating with the phase ramping from 0 to 2π in each grating period. An iris is placed at the intermediate image plane to control the field of view, enabling the discretization of k in Eq. 1. A polarizer is used to select the vertically polarized component to match the modulation polarization of the SLM. The final image is relayed by lens L3 ($f_{3}=500$ mm) and recorded using an EMCCD camera (iXon 885 K, Andor). For the DI image, we display a blank hologram on the SLM, so the SLM serves as a mirror.

**Fig. 2.**
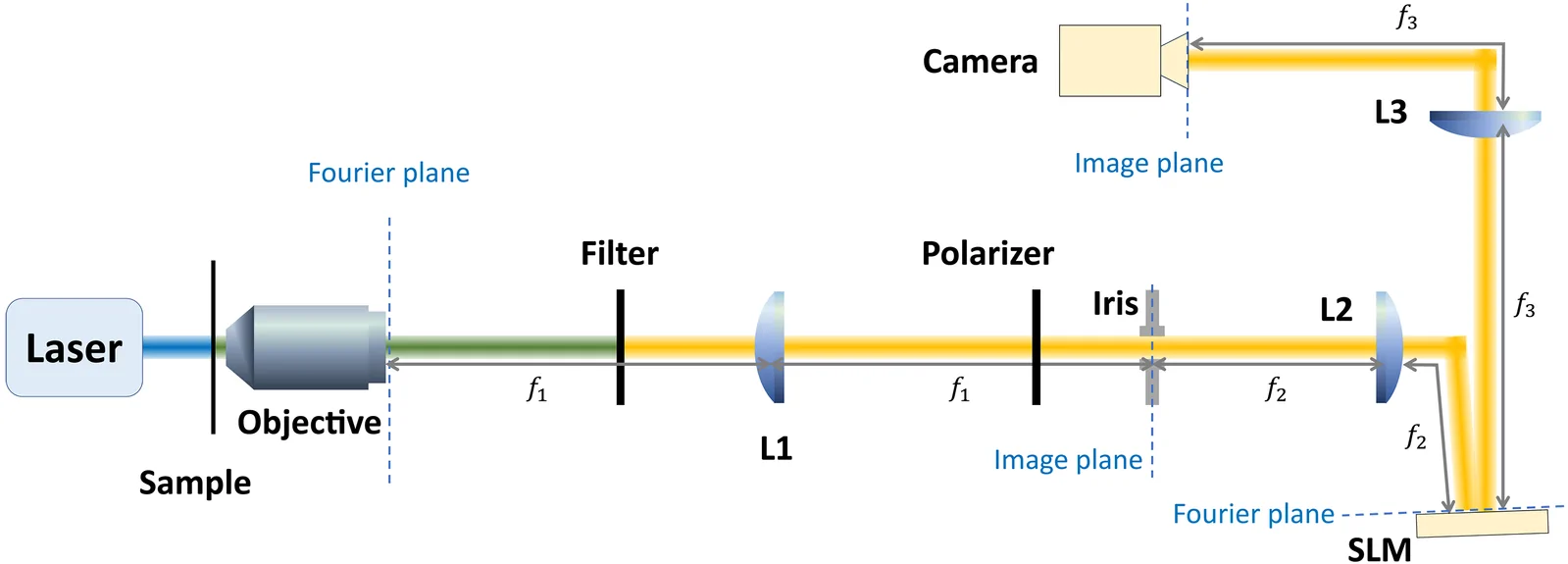
Experimental setup for FDD. A continuous-wave (CW) laser (473 nm) excites the fluorescent resolution test chart. The emitted fluorescence is collected by an oil-immersion objective (100×, NA = 1.4). A 4-f system consisting of lenses L1 and L2 images the Fourier plane onto the spatial light modulator (SLM), where the pupil function $h^{\left(l\right)}$ is displayed. An iris at the intermediate image plane limits the field of view. The final image is recorded by an EMCCD camera.

The field of view is ∼6 μm in the sample plane, corresponding to 2.7 mm on the camera. In principle, the five raw images can be acquired simultaneously by shifting four of them in four different directions while leaving the fifth image unshifted. However, in our proof-of-concept experiment, the five FDD raw images are acquired sequentially to reduce chromatic aberrations arising from the finite bandwidth of the filtered fluorescence. Specifically, to acquire the lth image, a constant phase is applied to the lth pupil function, while a linear phase ramp is applied to the other four pupil functions to deflect the unwanted light away from the optical axis. Simultaneous acquisition of all five raw images would be possible by using an achromatic phase modulation device. The phase gradient applied to the SLM is 60.8 rad/μm, corresponding to a spatial shift of 4.4 mm on the camera, which exceeds the image size (field of view) and thus prevents overlap of the images associated with different diffraction orders.

With NA = 1.4 and the fluorescent wavelength λ=540 nm, the cutoff frequency of our system $k_{c}=\frac{4\mathrm{\pi NA}}{\lambda }$ corresponds to 5184 lines/mm. The Rayleigh criterion $\left(\frac{0.61\lambda }{\text{NA}}=235\text{nm}\right)$ corresponds to 4255 lines/mm. The sample frequency of 4500 lines/mm is beyond the Rayleigh criterion but within the cutoff frequency. The raw images are cropped to 276 × 264 pixels, with the pixel size corresponding to 16.7 nm. Figure 3A compares FDD, DI, and deconvolved DI (DI dcv) images acquired with 244 photons per pixel (measured within the red circle). For the hybrid FDD measurement, the same total photon budget as for DI is maintained: approximately (244α=98) photons per pixel are allocated to the DI channel, while the remaining [244(1−α)=146] photons per pixel are distributed among the five FDD channels. The FDD and DI dcv images are reconstructed via Eqs. 9a-c and 10a-c, respectively, with three iterations. The DI and DI dcv images appear dominated by noise, the lines cannot be distinguished, and deconvolution amplifies the high frequency noise. In contrast, FDD under the same photon budget resolves the five parallel lines.

**Fig. 3.**
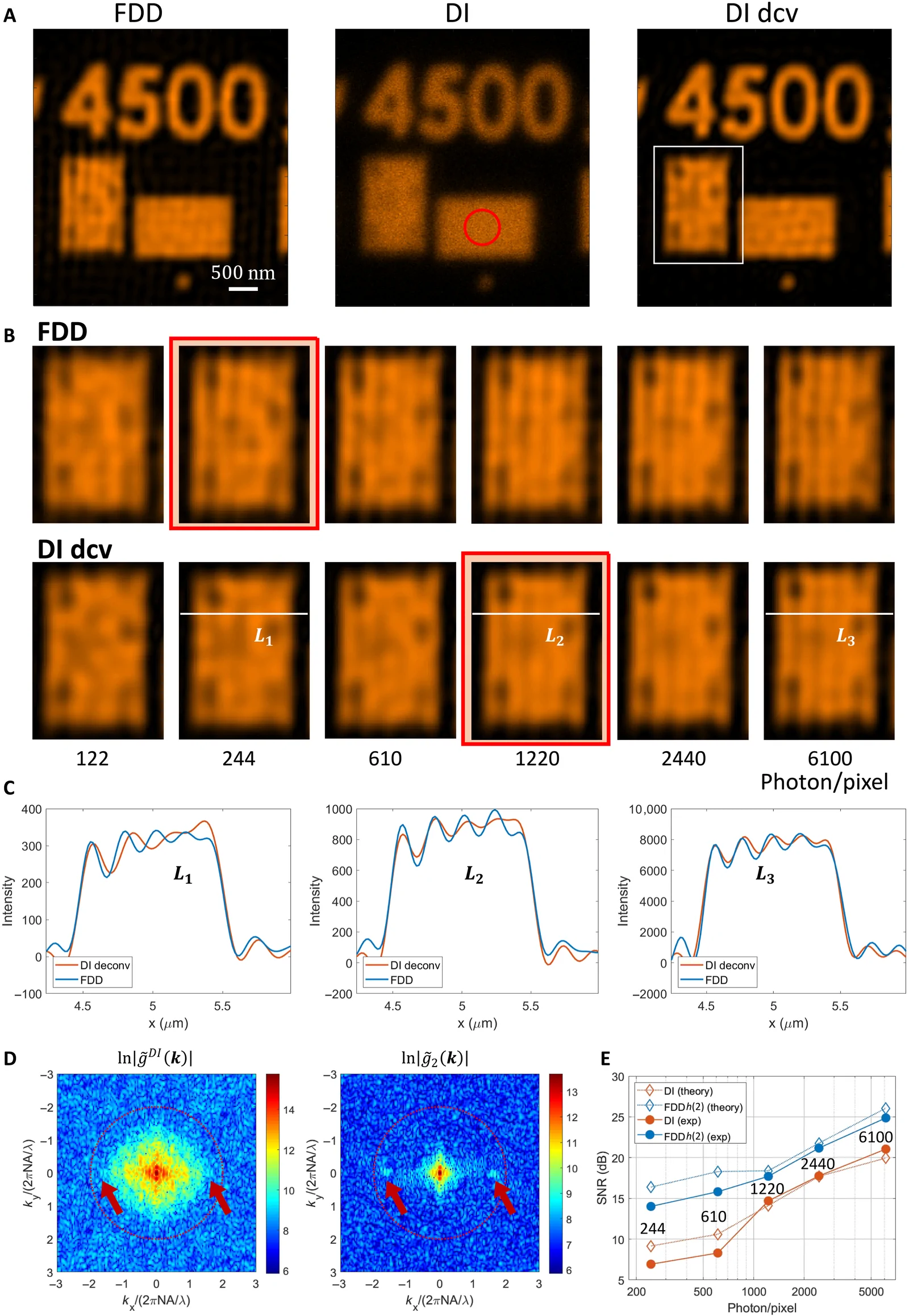
Experimental validation of FDD. (**A**) Comparison of FDD, DI, and DI deconvolution (DI dcv) for a fluorescent resolution chart (4500 lines/mm). Within the red circle, the average photon number is 244 photons/pixel. DI is dominated by noise, while FDD resolves five lines. (**B**) Zoomed-in regions (white box in A) at different photon budgets; FDD resolves the lines at 244 photons/pixel, whereas DI dcv requires ≥1220 photons per pixel (indicated by red boxes, respectively). (**C**) Line profiles along L_1_–L_3_ labeled in (B). (**D**) Fourier spectra of DI and FDD raw data (244 photon/pixel) of the second pupil $h_{2}$; high-frequency components (red arrows) are visible in FDD but buried in noise in DI. (**E**) SNR of the 4500 lines/mm component ($|k|=0.87k_{c}$) versus photon budget; FDD shows 4.97 dB improvement on average.

Notably, the horizontal line quintuplet is not resolved by any of the methods. This is because the vector nature of the field causes the PSF to become slightly wider in the dimension parallel to the polarization direction under a high-NA objective lens (*21*), which is vertical in our case.

A systematic comparison of FDD and DI dcv under different photon budgets is shown in Fig. 3 (B and C), where the region in the white box in Fig. 3A is selected for comparison. For FDD, 244 photons per pixel are sufficient to resolve the five-line structure (indicated by the red box), whereas DI dcv requires at least 1220 photons per pixel to achieve comparable resolution (indicated by the red box). At high photon budget, both methods recover the five lines with similar quality.

Figure 3D illustrates the advantage of FDD in the Fourier domain. The red arrows indicate high-frequency components corresponding to the 4500 lines/mm structure. In the FDD data from the second pupil ($h_{2}$) (Fig. 3D, right), these components are visible, whereas in the DI case (Fig. 3D, left), they are buried in noise due to the higher shot noise level (brighter background). Last, the SNR of the Fourier component at 4500 lines/mm is plotted in Fig. 3E as a function of photon per pixel, which is labeled in the figure. The noise of a raw image was quantified in two ways: (i) theoretically, using the shot-noise model $\langle {|{\tilde{n}}_{l}\left(k\right)|}^{2}\rangle =N_{l}$ (eq. S28 in section S3), where $N_{l}$ is the total number of photons in the *l*th pupil; (ii) experimentally, by averaging the intensity outside the cutoff frequency in the Fourier domain, where no signal is present (Fig. 3D), according to $\langle {|{\tilde{n}}_{l}\left(k\right)|}^{2}\rangle =\frac{\int_{k>k_{c}}{|{\tilde{g}}_{l}\left(k\right)|}^{2}d^{2}k}{\int_{k>k_{c}}d^{2}k}$. Compared with DI, FDD should theoretically achieve an SNR improvement of 6.63 dB, on average, for $|k|∼k_{b}$. In experiment, the SNR improves by 4.97 dB. The discrepancy is due to electronic noise.

## DISCUSSION

To summarize, FDD, guided by quantum estimation theory, achieves a measurement advantage over DI under photon-starved conditions. In DI, the light transmitted through the entire pupil is recorded as a single image. The contributions from different spatial frequencies are then mixed together, and the weak signatures of high-frequency Fourier components are largely overwhelmed by the shot noise from much stronger low-frequency components, resulting in poor estimation precision. FDD addresses this problem by detecting the images generated by several subsets of high spatial frequencies separately, thus enhancing the precision of estimating their amplitudes.

FDD shows substantial advantage with respect to DI in terms of photon budget, making it ideal for imaging scenarios under limited photon numbers. Instances include photo-toxicity in bioimaging (*22*), observation of faint celestial bodies in astronomy, and high speed photography with extremely short exposure times. In addition, because FDD modifies only the detection optics through passive optical elements, without requiring active manipulation of either the sample or the illumination, the proposed approach is, in principle, applicable to a broad range of incoherent imaging systems. This makes it particularly attractive for applications where active interaction with the object is impractical or impossible, such as astronomical imaging, satellite imaging, surveillance, and remote sensing in the photon-starved regime.

A potentially perplexing feature of FDD is the relatively modest resolution enhancement beyond the diffraction limit, despite substantial improvement of the SNR. To understand this, we recall that to substantially enhance resolution, we would need access to spatial frequency components beyond cutoff, which is not possible with passive optics without a priori information about the object. However, this is not the case here. Both FDD and DI have access to the same set of Fourier components—those with $|k|\leq k_{c}$. FDD only enhances the measurement precision of the components just below the cutoff and hence improves the deconvolution accuracy in the photon-starved regime.

The novelty of our method stems from its foundation in quantum estimation theory. By analytically calculating QFI and FI, we convert the quantum optimization task to a classical OTF engineering problem—more specifically, optimizing the ratio β(k)/β(0). This enables relatively straightforward extension of our approach to other imaging modalities, beyond incoherent linear imaging under wide-field illumination studied here.

1) For nonlinear imaging methods, such as two-photon fluorescence, the OTF deviates from a triangular shape, exhibiting a sharp peak around zero and quickly falling off for higher frequency components (*23*). Hence, we expect that FDD, which reduces the denominator β(0), will be even more beneficial than in the linear case. The expected resolution improvement can be more than two times (*24*).

2) Prior knowledge of the sample, e.g., limited size, helps to retrieve the frequency components beyond the cutoff. However, the accuracy of the retrieval depends on the SNR (*25*). Since our method improves the SNR substantially for high frequency components close to the cutoff, it is interesting to explore the QFI, the FI, and the optimal detection in this scenario.

3) If a structured pattern of illumination is an option, then this gives us another degree of freedom, which affects the quantum state of light entering the objective lens, and hence its QFI. By optimizing the illumination structure in concert with the OTF, further resolution enhancements can be expected.

4) The analysis presented here follows the Tsang framework (*4*) in approximating the state of light collected in each temporal mode as a mixture of vacuum and single-photon contributions. Extending quantum super-resolution beyond this weak source regime has been the subject of substantial recent work, both in the derivation of quantum bounds valid for arbitrary source strengths and general field states (*8*, *26*) and in schemes that exploit the nonclassical photon statistics of quantum emitters to enhance resolution (*27*, *28*). Adapting FDD to this generalized field statistics requires reformulating the quantum measurement model and is an interesting direction for future work.

5) The present analysis is based on FI, which provides an asymptotic characterization of estimation precision. In the finite-sample regime, however, estimator bias and higher-order statistical effects can become non-negligible, where FI no longer provides a complete characterization of the achievable estimation performance. Recent work has developed Bayesian frameworks (*29*), higher-order asymptotic corrections (*30*, *31*), and sample-complexity approaches (*32*) to address this limitation. We analyze the bias and variance of the estimator used in this work (see section S3), showing that it is asymptotically unbiased and asymptotically attains the CRB at large *N*. At low photon numbers, however, the estimator becomes biased, so the standard CRB for unbiased estimators is no longer directly applicable.

Although FDD surpasses the DI for high spatial frequency components, its CRB remains more than a factor of two above the QCRB (Fig. 1E). An important question is whether this gap can be closed. Although the estimation problem is multiparameter, the symmetric logarithmic derivative operators approximately satisfy the weak commutativity condition (see section S1), implying that the QCRB approximately coincides with the Holevo bound and is therefore asymptotically attainable (*33*, *34*).

This being said, the Holevo bound is attainable only if collective (entangling) measurements over asymptotically many copies of the state are allowed. On the other hand, linear optics followed by intensity detection with thermal input states corresponds to single-copy (separable) measurements on individual photons. Under these condition, the uncertainty is bounded by the Nagaoka-Hayashi Cramér-Rao bound (*35*). The calculation of this bound and its practical attainability is deferred to future research.

## Supplementary Materials

### This PDF file includes:

Sections S1 to S3 Fig. S1 References
