# Supplementary material for: Passive imaging with quantum advantage
Source: Sci Adv. 2026 Nov 11;12(46):eaei1896. doi: 10.1126/sciadv.aei1896 (PMC13754014; doi:10.1126/sciadv.aei1896)
Supplement: Supplementary file 1 [file sciadv.aei1896_sm.pdf]

Science Advances
AAAS

Supplementary Materials for

Passive imaging with quantum advantage

Li Gong *et al.*

Corresponding author: Li Gong, gong_li@a-star.edu.sg

Sci. Adv. **12**, eaei1896 (2026)
DOI: 10.1126/sciadv.aei1896

This PDF file includes:

Sections S1 to S3
Fig. S1
References

Supplementary Materials for Passive Imaging with Quantum Advantage

S1. QFI and FI analysis.

In this subsection, we derive approximate analytical solutions for the QFI, the FI of DI, and the FI of FDD. These analytical results are then validated by comparing with numerical solutions obtained without approximation.

Quantum Fisher information

The quantum Fisher information per photon can be calculated as (36)

$$\mathrm{QFI}_{ij} = \mathrm{Re}\left[\mathrm{tr}\left(\hat{\rho}_1 \hat{L}_i \hat{L}_j\right)\right], \tag{S1}$$

where

$$\frac{\partial \hat{\rho}_1}{\partial \theta_i} = \frac{1}{2}\left(\hat{\rho}_1 \hat{L}_i + \hat{L}_i \hat{\rho}_1\right). \tag{S2}$$

defines the SLD $\hat{L}_i$. These equations can be solved analytically under the *low-contrast approximation* $a_{i\neq 0}, b_{i\neq 0} \ll a_0$, in which case we can neglect nonzero spatial frequency components in the right-hand side of Eq. (S2), replacing the density operator by

$$\hat{\rho}_1^{(\mathrm{appr})} = \iint a_0 e^{-i\hat{\mathbf{K}}\cdot\mathbf{r}} \ |\Psi\rangle\langle\Psi| \ e^{i\hat{\mathbf{K}}\cdot\mathbf{r}} d^2\mathbf{r}. \tag{S3}$$

Using the original density matrix for the left-hand side, we find

$$\frac{\partial \hat{\rho}_1}{\partial a_{\mathbf{k}}} = \iint \cos\left(\mathbf{k}\cdot\mathbf{r}\right) e^{-i\hat{\mathbf{K}}\cdot\mathbf{r}} \ |\Psi\rangle\langle\Psi| \ e^{i\hat{\mathbf{K}}\cdot\mathbf{r}} d^2\mathbf{r} \tag{S4a}$$

$$\frac{\partial \hat{\rho}_1}{\partial b_{\mathbf{k}}} = \iint \sin\left(\mathbf{k}\cdot\mathbf{r}\right) e^{-i\hat{\mathbf{K}}\cdot\mathbf{r}} \ |\Psi\rangle\langle\Psi| \ e^{i\hat{\mathbf{K}}\cdot\mathbf{r}} d^2\mathbf{r}. \tag{S4b}$$

Then we obtain the approximation of SLD as

$$\hat{L}_i^{(\mathrm{appr})} = \frac{k_c^2}{16\pi a_0}\frac{\partial \hat{\rho}_1}{\partial \theta_i}. \tag{S5}$$

The derivation of this result is tedious. However, it can be verified as follows. From Eqs. (S3) and (S5) we have

$$\begin{aligned}
\hat{\rho}_1^{(\mathrm{appr})} \hat{L}_{a_{\mathbf{k}}}^{(\mathrm{appr})} &= \frac{k_c^2}{16\pi a_0} \iint a_0 e^{-i\hat{\mathbf{K}}\cdot\mathbf{r}}|\Psi\rangle\langle\Psi| e^{i\hat{\mathbf{K}}\cdot\mathbf{r}} d^2\mathbf{r} \\
&\times \iint \cos\left(\mathbf{k}\cdot\mathbf{r}'\right) e^{-i\hat{\mathbf{K}}\cdot\mathbf{r}'}|\Psi\rangle\langle\Psi| e^{i\hat{\mathbf{K}}\cdot\mathbf{r}'} d^2\mathbf{r}'. \\
&= \frac{k_c^2}{16\pi} \iint d^2\mathbf{r}' \cos(\mathbf{k}\cdot\mathbf{r}') \\
&\times \left[\iint d^2\mathbf{r} \left\langle \Psi | e^{i\hat{\mathbf{K}}\cdot\mathbf{r}} e^{-i\hat{\mathbf{K}}\cdot\mathbf{r}'} |\Psi\right\rangle e^{-i\hat{\mathbf{K}}\cdot\mathbf{r}}|\Psi\rangle\right] \langle\Psi| e^{i\hat{\mathbf{K}}\cdot\mathbf{r}'}
\end{aligned} \tag{S6}$$

The term in angular brackets in the last line is a self-convolution of the APSF:

$$\begin{aligned}
\left\langle \Psi | e^{i\hat{\mathbf{K}}\cdot\mathbf{r}} e^{-i\hat{\mathbf{K}}\cdot\mathbf{r}'} |\Psi\right\rangle &= \iint \langle\Psi| e^{i\hat{\mathbf{K}}\cdot\mathbf{r}} |\mathbf{r_0}\rangle\langle\mathbf{r_0}| e^{-i\hat{\mathbf{K}}\cdot\mathbf{r}'}|\Psi\rangle d^2\mathbf{r_0} \\
&= \iint \Psi(\mathbf{r}-\mathbf{r_0})\Psi(\mathbf{r}'-\mathbf{r_0}) d^2\mathbf{r_0} = \frac{4\sqrt{\pi}}{k_c}\Psi(\mathbf{r}-\mathbf{r}').
\end{aligned} \tag{S7}$$

In the last equality, we used the fact that $\Psi(\mathbf{r}) = \frac{J_1(k_c\mathbf{r}/2)}{\sqrt{\pi}\mathbf{r}}$ is a normalized Airy disk, so its self convolution is also an Airy disk, i.e., $\iint \Psi(\mathbf{r})\Psi(\mathbf{r}'-\mathbf{r}) d^2\mathbf{r} = \frac{4\sqrt{\pi}}{k_c}\Psi(\mathbf{r}')$. This is because the Fourier transform of the Airy disk is a top-hat function, and its self-product is also a top-hat function.

Hence

$$
\begin{aligned}
\hat{\rho}_1^{(\mathrm{appr})}\hat{L}_{a_{\mathbf{k}}}^{(\mathrm{appr})} &= \frac{k_c^2}{16\pi}\iint d^2\mathbf{r}'\cos(\mathbf{k}\cdot\mathbf{r}')\left[\iint d^2\mathbf{r}\frac{4\sqrt{\pi}}{k_c}\Psi(\mathbf{r}-\mathbf{r}')e^{-i\hat{\mathbf{K}}\cdot\mathbf{r}}\ \ |\Psi\rangle\right]\langle\Psi|e^{i\hat{\mathbf{K}}\cdot\mathbf{r}'} \\
&= \frac{k_c^2}{16\pi}\iint d^2\mathbf{r}'\cos(\mathbf{k}\cdot\mathbf{r}')\left[\iint d^2\mathbf{r}\frac{4\sqrt{\pi}}{k_c}\Psi(\mathbf{r}-\mathbf{r}')\iint d^2\mathbf{r_0}|\mathbf{r_0}\rangle\langle\mathbf{r_0}|e^{-i\hat{\mathbf{K}}\cdot\mathbf{r}}\ \ |\Psi\rangle\right]\langle\Psi|e^{i\hat{\mathbf{K}}\cdot\mathbf{r}'} \\
&= \frac{k_c^2}{16\pi}\iint d^2\mathbf{r}'\cos(\mathbf{k}\cdot\mathbf{r}')\left[\frac{4\sqrt{\pi}}{k_c}\iint d^2\mathbf{r_0}|\mathbf{r_0}\rangle\iint d^2\mathbf{r}\Psi(\mathbf{r}-\mathbf{r}')\Psi(\mathbf{r}-\mathbf{r_0})\right]\langle\Psi|e^{i\hat{\mathbf{K}}\cdot\mathbf{r}'} \\
&= \iint d^2\mathbf{r}'\cos(\mathbf{k}\cdot\mathbf{r}')\left[\iint d^2\mathbf{r_0}|\mathbf{r_0}\rangle\Psi(\mathbf{r}'-\mathbf{r_0})\right]\langle\Psi|e^{i\hat{\mathbf{K}}\cdot\mathbf{r}'} \\
&= \iint d^2\mathbf{r}'\cos(\mathbf{k}\cdot\mathbf{r}')e^{-i\hat{\mathbf{K}}\cdot\mathbf{r}'}\ \ |\Psi\rangle\langle\Psi|e^{i\hat{\mathbf{K}}\cdot\mathbf{r}'} = \frac{\partial\hat{\rho}_1}{\partial a_{\mathbf{k}}},
\end{aligned}
\tag{S8}
$$

where we again used the aforementioned identity for the self-convolution of the Airy disk.

Similarly, we have $\hat{L}_{a_{\mathbf{k}}}^{(\mathrm{appr})}\hat{\rho}_1^{(\mathrm{appr})} = \frac{\partial\hat{\rho}_1}{\partial a_{\mathbf{k}}}$. Therefore, we have verified that the SLD $\hat{L}_{a_{\mathbf{k}}}^{(\mathrm{appr})}$ in Eq. (S5) is a solution of Eq. (S2). The same is true for the sin mode $\hat{L}_{b_{\mathbf{k}}}^{(\mathrm{appr})}$. Then, by replacing $\hat{L}_i$ with $\hat{L}_i^{(\mathrm{appr})}$ and $\hat{\rho}_1$ with $\hat{\rho}_1^{(\mathrm{appr})}$ in Eq. (S1) , we obtain

$$
\begin{aligned}
&\mathrm{QFI}_{a_{\mathbf{k}},a_{\mathbf{k}}} \approx \mathrm{Re}\left[\mathrm{tr}\left(\hat{\rho}_1^{(\mathrm{appr})}\hat{L}_{a_{\mathbf{k}}}^{(\mathrm{appr})}\hat{L}_{a_{\mathbf{k}}}^{(\mathrm{appr})}\right)\right] \\
&= \frac{k_c^2}{16\pi a_0}\mathrm{Re}\left[\mathrm{tr}\left(\frac{\partial\hat{\rho}_1}{\partial a_{\mathbf{k}}}\frac{\partial\hat{\rho}_1}{\partial a_{\mathbf{k}}}\right)\right] \\
&= \frac{1}{a_0}\iint d^2\mathbf{r}\iint d^2\mathbf{r}'\cos(\mathbf{k}\cdot\mathbf{r})\cos(\mathbf{k}\cdot\mathbf{r}')\left[\Psi(\mathbf{r}-\mathbf{r}')\right]^2 \\
&= \frac{\beta^{\mathrm{DI}}(\mathbf{k})}{a_0}\iint d^2\mathbf{r}\cos^2(\mathbf{k}\cdot\mathbf{r}) \\
&= \frac{\beta^{\mathrm{DI}}(\mathbf{k})}{2a_0^2}.
\end{aligned}
\tag{S9}
$$

In the last two steps, we have used $\iint d^2\mathbf{r}'\cos(\mathbf{k}\cdot\mathbf{r}')\left[\Psi(\mathbf{r}-\mathbf{r}')\right]^2 = \beta^{\mathrm{DI}}(\mathbf{k})\cos(\mathbf{k}\cdot\mathbf{r})$ and $\iint d^2\mathbf{r}\cos^2(\mathbf{k}\cdot\mathbf{r}) = \frac{A}{2}$, where A is the area of the sample. The normalization condition $\iint f(\mathbf{r})\ d^2\mathbf{r} = 1$ leads to $A = 1/a_0$. Similar procedures show that the diagonal elements for sine modes $\mathrm{QFI}_{b_{\mathbf{k}},b_{\mathbf{k}}} \approx \mathrm{QFI}_{a_{\mathbf{k}},a_{\mathbf{k}}}$, because $\iint d^2\mathbf{r}\sin^2(\mathbf{k}\cdot\mathbf{r}) = \frac{A}{2}$ as well. There are two types of off-diagonal elements in QFI matrix: (1) interaction between cosine and sine modes, i.e., $\mathrm{QFI}_{a_{\mathbf{k}},b_{\mathbf{k}'}}$ and $\mathrm{QFI}_{b_{\mathbf{k}'},a_{\mathbf{k}}}$, which are proportional to $\iint d^2\mathbf{r}\cos(\mathbf{k}\cdot\mathbf{r})\sin(\mathbf{k}'\cdot\mathbf{r}) = 0$; (2) interaction between two cosine and sine modes with different frequencies, i.e., $\mathrm{QFI}_{a_{\mathbf{k}},a_{\mathbf{k}'}}$ and $\mathrm{QFI}_{b_{\mathbf{k}},b_{\mathbf{k}'}}$ with $\mathbf{k} \neq \mathbf{k}'$, which are proportional to $\iint d^2\mathbf{r}\cos(\mathbf{k}\cdot\mathbf{r})\cos(\mathbf{k}'\cdot\mathbf{r}) = \iint d^2\mathbf{r}\sin(\mathbf{k}\cdot\mathbf{r})\sin(\mathbf{k}'\cdot\mathbf{r}) = 0$. As a result, we can express the approximate solution of QFI matrix as Eq. (3).

Weak commutativity condition

In the same vein as Eq. (S9), we can see that $\mathrm{tr}\left(\hat{\rho}_1^{(\mathrm{appr})}\hat{L}_{a_{\mathbf{k}}}^{(\mathrm{appr})}\hat{L}_{a_{\mathbf{k}'}}^{(\mathrm{appr})}\right) = \mathrm{tr}\left(\hat{\rho}_1^{(\mathrm{appr})}\hat{L}_{b_{\mathbf{k}}}^{(\mathrm{appr})}\hat{L}_{b_{\mathbf{k}'}}^{(\mathrm{appr})}\right) = 0$ for $\mathbf{k} \neq \mathbf{k}'$, because these terms are proportional to $\iint d^2\mathbf{r}\cos(\mathbf{k}\cdot\mathbf{r})\cos(\mathbf{k}'\cdot\mathbf{r}) = \iint d^2\mathbf{r}\sin(\mathbf{k}\cdot\mathbf{r})\sin(\mathbf{k}'\cdot\mathbf{r}) = 0$. Similarly, $\mathrm{tr}\left(\hat{\rho}_1^{(\mathrm{appr})}\hat{L}_{a_{\mathbf{k}}}^{(\mathrm{appr})}\hat{L}_{b_{\mathbf{k}'}}^{(\mathrm{appr})}\right) = 0$, because $\iint d^2\mathbf{r}\cos(\mathbf{k}\cdot\mathbf{r})\sin(\mathbf{k}'\cdot\mathbf{r}) = 0$. Therefore, we conclude that $\mathrm{tr}\left(\hat{\rho}_1^{(\mathrm{appr})}\hat{L}_i^{(\mathrm{appr})}\hat{L}_j^{(\mathrm{appr})}\right) = 0$, for $i \neq j$. Hence,

$$
\begin{aligned}
&\mathrm{tr}\left(\hat{\rho}_1\left[\hat{L}_i,\hat{L}_j\right]\right) \\
&\approx \mathrm{tr}\left(\hat{\rho}_1^{(\mathrm{appr})}\left[\hat{L}_i^{(\mathrm{appr})}\hat{L}_j^{(\mathrm{appr})} - \hat{L}_j^{(\mathrm{appr})}\hat{L}_i^{(\mathrm{appr})}\right]\right) \\
&= 0
\end{aligned}
\tag{S10}
$$

for $i \neq j$. In this regard, the weak commutativity condition (34) is approximately satisfied, owing to the orthogonality of the Fourier modes — meaning that the QCRB approximately coincides with the Holevo bound (33,34) and is thus asymptotically attainable for the present multiparameter estimation problem.

Fisher information for DI and FDD

With the total photon number N and normalized ground truth $f(\mathbf{r})$, the recorded photon density in the DI image is $g^{\mathrm{DI}}(\mathbf{r}) = \langle g^{\mathrm{DI}}(\mathbf{r})\rangle + n^{\mathrm{DI}}(\mathbf{r})$, where $\langle g^{\mathrm{DI}}(\mathbf{r})\rangle$ is the mean value, $n^{\mathrm{DI}}(\mathbf{r})$ is the noise. In the Fourier domain, $\tilde{g}^{\mathrm{DI}}(\mathbf{k}) = \langle\tilde{g}^{\mathrm{DI}}(\mathbf{k})\rangle + \tilde{n}^{\mathrm{DI}}(\mathbf{k})$, with $\langle\tilde{g}^{\mathrm{DI}}(\mathbf{k})\rangle =$

$N\beta^{\mathrm{DI}}(\mathbf{k})\,\widetilde{f}(\mathbf{k})$. Thus, in spatial domain, it is

$$\langle g^{\mathrm{DI}}(\mathbf{r})\rangle = N\beta^{\mathrm{DI}}(0)a_0 \tag{S11}$$

$$+ N \sum_{\substack{|\mathbf{k}|\le k_c \\ k_x,k_y>0}} \beta^{\mathrm{DI}}(\mathbf{k})\left[a_{\mathbf{k}}\cos(\mathbf{k}\cdot\mathbf{r}) + b_{\mathbf{k}}\sin(\mathbf{k}\cdot\mathbf{r})\right] \tag{S12}$$

Then, the mean photon number on the ith pixel at $\mathbf{r}_i$ is $\langle g^{\mathrm{DI}}(\mathbf{r}_i)\rangle\Delta x^2$, where Δx^2 is the area of the pixel. Here we assume that the pixel size of the detector is much smaller than the size of the PSF. Assuming the detection is limited by the shot noise, which follows the Poisson distribution, the classical FI per photon of DI can be calculated as (4)

$$\mathrm{FI}^{\mathrm{DI}}_{ij} = \frac{1}{N}\iint \frac{1}{\langle g^{\mathrm{DI}}(\mathbf{r})\rangle}\frac{\partial\langle g^{\mathrm{DI}}(\mathbf{r})\rangle}{\partial\theta_i}\frac{\partial\langle g^{\mathrm{DI}}(\mathbf{r})\rangle}{\partial\theta_j}d^2\mathbf{r}, \tag{S13}$$

In this integral, $\frac{\partial\langle g^{\mathrm{DI}}(\mathbf{r})\rangle}{\partial\theta_i}$ can be evaluated exactly as

$$\frac{\partial\langle g^{\mathrm{DI}}(\mathbf{r})\rangle}{\partial a_{\mathbf{k}}} = N\beta^{\mathrm{DI}}(\mathbf{k})\cos(\mathbf{k}\cdot\mathbf{r})\,, \tag{S14a}$$

$$\frac{\partial\langle g^{\mathrm{DI}}(\mathbf{r})\rangle}{\partial b_{\mathbf{k}}} = N\beta^{\mathrm{DI}}(\mathbf{k})\sin(\mathbf{k}\cdot\mathbf{r})\,, \tag{S14b}$$

while $\frac{1}{\langle g^{\mathrm{DI}}\rangle}$ can be evaluated approximately as $\frac{1}{N\beta^{\mathrm{DI}}(0)a_0}$. This approximation is a classical version of the approximation applied to the calculation of QFI above. Then the FI of DI can be calculated analytically as

$$\begin{aligned}\mathrm{FI}^{\mathrm{DI}}_{a_{\mathbf{k}},a_{\mathbf{k}}} &\approx \frac{1}{N}\iint \frac{1}{N\beta^{\mathrm{DI}}(0)a_0}\left[N\beta^{\mathrm{DI}}(\mathbf{k})\cos(\mathbf{k}\cdot\mathbf{r})\right]^2 d^2\mathbf{r}, \\ &= \frac{\left[\beta^{\mathrm{DI}}(\mathbf{k})\right]^2}{\beta^{\mathrm{DI}}(0)a_0}\iint \cos^2(\mathbf{k}\cdot\mathbf{r})\,d^2\mathbf{r} \\ &= \frac{\left[\beta^{\mathrm{DI}}(\mathbf{k})\right]^2}{2\beta^{\mathrm{DI}}(0)a_0^2}\end{aligned} \tag{S15}$$

Again, in the last step, we have used $\iint\cos^2(\mathbf{k}\cdot\mathbf{r})\,d^2\mathbf{r} = \frac{A}{2} = \frac{1}{2a_0}$. Since $\beta^{\mathrm{DI}}(0) = 1$, $\mathrm{FI}^{\mathrm{DI}}_{a_{\mathbf{k}},a_{\mathbf{k}}} = \frac{\left[\beta^{\mathrm{DI}}(\mathbf{k})\right]^2}{2a_0^2}$. Similarly, the diagonal element for the sin mode is $\mathrm{FI}^{\mathrm{DI}}_{b_{\mathbf{k}},b_{\mathbf{k}}} = \frac{\left[\beta^{\mathrm{DI}}(\mathbf{k})\right]^2}{2a_0^2}$ as well. All the off-diagonal elements are zero. Hence, we can express FI matrix as Eq. (4).

The FI of FDD for the lth pupil function h_l can be calculated via the same procedure from Eq. (S12) to Eq. (S15), by replacing β^{DI} with β_l, resulting in Eq. (5).

Numerical calculation of quantum and classical FI

The analytical results of QFI and FI have been obtained with an approximation. In order to verify the validity of the approximations employed, we compare the analytical results with numerical solutions obtained without approximation. The numerical FI for the DI case can be computed using Eqs. (S12)–(S13) above. For the FDD case, the same equations apply with β^{DI} replaced by β_l. The numerical QFI can be evaluated as follows. Given the spectral decomposition of the single-photon density matrix

$$\hat{\rho}_1 = \sum_{l\in S}\xi_l\,|\psi_l\rangle\langle\psi_l|, \tag{S16}$$

where $S = \{l\,|\,\xi_l \neq 0\}$ is the support, an element of the QFI can be calculated as (36)

$$\begin{aligned}\mathrm{QFI}_{ij} = &\sum_{l\in S}\frac{(\partial_i\xi_l)(\partial_j\xi_l)}{\xi_l} + \sum_{l\in S}4\xi_l\,\mathrm{Re}(\langle\partial_i\psi_l|\partial_j\psi_l\rangle) \\ &- \sum_{l,m\in S}\frac{8\xi_l\xi_m}{\xi_l+\xi_m}\mathrm{Re}(\langle\partial_i\psi_l|\psi_m\rangle\langle\psi_m|\partial_j\psi_l\rangle)\,.\end{aligned} \tag{S17}$$

We compare the analytical and numerical QFI calculations for a one-dimensional sample, such that $f(x) = a_0 + \sum_l a_l\cos(k_l x) + b_l\sin(k_l x)$, where a_l and b_l $(1 \le l \le 32)$ are random numbers sampled from a Gaussian distribution, with a_0 chosen to make $f(x)$ positive. Figs. S1(e,f) display the ground truth intensity profiles for two such random samples. Sample 1 [Fig. S1(e)] with the coefficient of variation $R = \mathrm{std}[f(x)]/a_0 = 4.43\%$ represents a nearly uniform sample, while sample 2 [Fig. S1(f)] with $R = 56.03\%$ is highly random. Figs. S1(a,c) display the numerically computed QFI, FI (DI), and FI (FDD hybrid) matrices for these two samples, while Figs. S1(b,d), show comparison between the numerically and analytically calculated QCRB and CRB matrices. We see that all the numerically computed QFI and FI matrices are nearly diagonal, consistent with the analytical results in which the corresponding matrices are exactly diagonal. The behavior of cosine (a_l) and sine (b_l) modes are almost the same, consistent with the analytical prediction where they are exactly the same.

To further evaluate the accuracy of the approximation, multiple random samples with different R are generated. For each sample, the analytical and numerical results are compared over the whole spatial frequency range, and the error, defined as the root mean square of the relative difference (analytical CRB − numerical CRB)/numerical CRB over all spatial frequencies, is plotted as a function of R in Fig. S1(g). As expected, the approximation error increases with R. However, even for highly random samples with large R, the error remains below 10%, demonstrating that the proposed approximations remain accurate over a broad range of sample inhomogeneity. The error is not exactly zero for $R = 0$ because finite number of Fourier modes result in periodic sample function, and in numerical solution the sample is truncated to a single period.

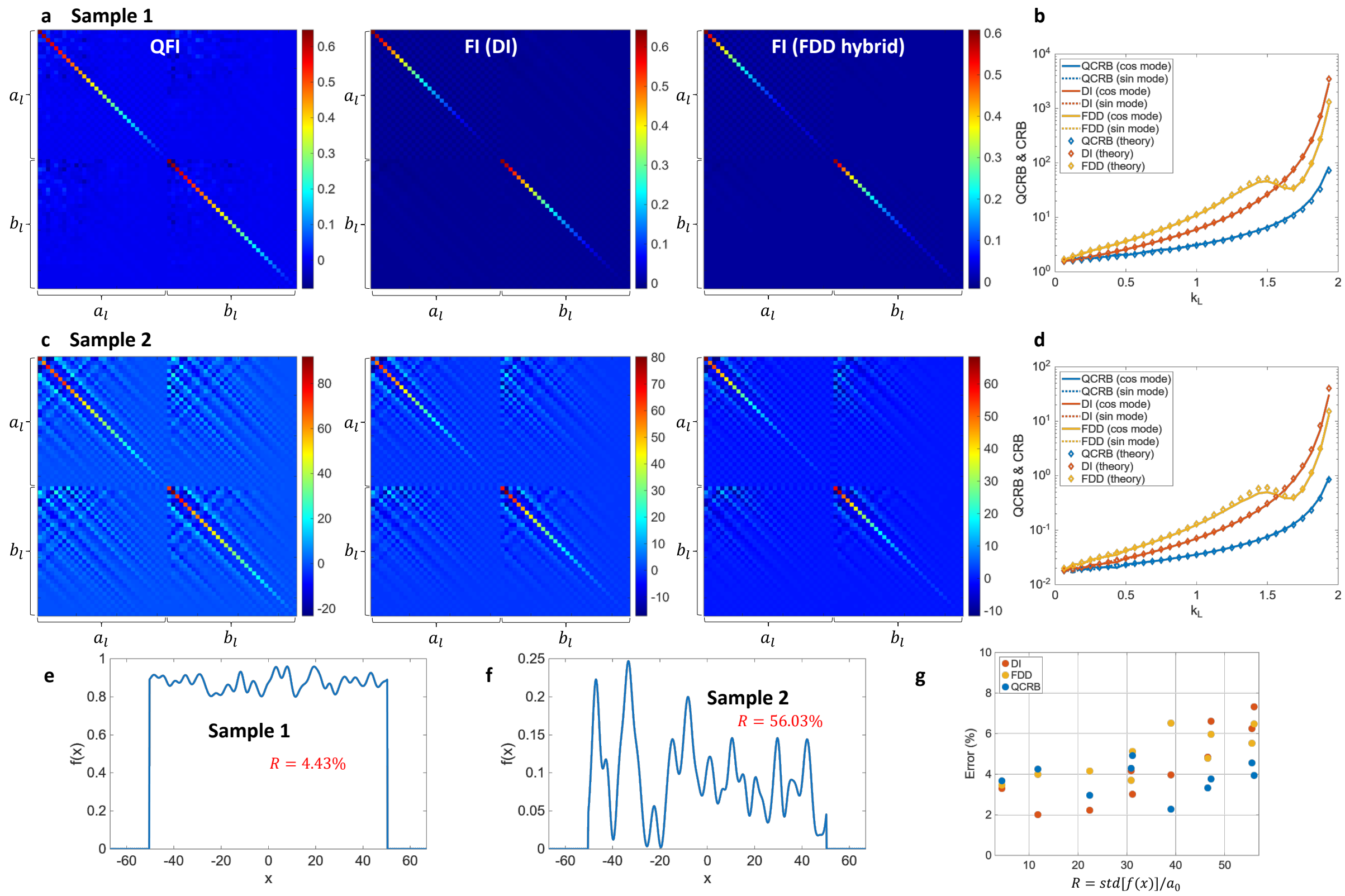

FIG. S1. **Comparison between approximate analytical and numerical results of QFI and FI. a, c**, numerically computed QFI, FI (DI), and FI (FDD hybrid) for sample 1 and sample 2, respectively. The matrices are nearly diagonal, consistent with the approximate analytical solutions, which are strictly diagonal. **b, d**, Comparison of the numerical QCRB and CRBs for cosine (solid curve) and sine (dashed curve) modes with the analytical results (diamonds) for sample 1 and sample 2 respectively. **e, f**, Intensity profiles for sample 1 and sample 2, respectively. **g**, Error versus coefficient of variation R for a large ensemble of random samples. The error is defined as the root mean square (over spatial frequency) of the relative difference between the analytical and numerical results. Each point corresponds to one random sample. The small error ($< 10\%$) demonstrates that the approximation remains accurate even for strongly inhomogeneous samples.

S2. Minimum photon number for achievable resolution

To derive Eq. (8), we assume that the variance of the parameter saturates the CRB, i.e.,

$$\langle \Delta\theta_i^2 \rangle = \frac{CRB_{i,i}}{N} \tag{S18}$$

For DI (Eq. (4)), the per-photon $\mathrm{CRB}_{ii}^{\mathrm{DI}} = \frac{2\beta^{\mathrm{DI}}(0)a_0^2}{[\beta^{\mathrm{DI}}(\mathbf{k_i})]^2}$, so achieving the signal-to-noise threshold $\gamma = \min_i \theta_i/\sqrt{\Delta\theta_i^2}$ requires

$$\frac{\theta_i^2}{\langle \Delta\theta_i^2 \rangle} = \frac{\theta_i^2\left[\beta^{\mathrm{DI}}(\mathbf{k_i})\right]^2}{2N\beta^{\mathrm{DI}}(0)a_0^2} \geq \gamma^2, \tag{S19}$$

Thus, the minimum total photon number is

$$N_{\min}^{\mathrm{DI}} = \max_{|\mathbf{k}_i|<k} \frac{2a_0^2}{\theta_i^2}\frac{\beta^{\mathrm{DI}}(0)}{\left[\beta^{\mathrm{DI}}(\mathbf{k}_i)\right]^2}\gamma^2. \tag{S20}$$

This number is inconvenient for two reasons: it scales with the sample area and we have no information about individual Fourier coefficients θ_i. To address the latter point, we replace in Eq. (S23) θ_i^2 by $\bar{\sigma}^2 \equiv \langle\theta_i^2\rangle_{\mathrm{k}}$ (i.e. assuming a white spectrum of $f(\mathbf{r})$. The parameter $\bar{\sigma}$ can be related to the properties of the sample as follows.

The variance of the sample function can be calculated as

$$\begin{aligned}\langle \Delta f(\mathbf{r})^2\rangle &= \langle f(\mathbf{r})^2\rangle - \langle f(\mathbf{r})\rangle^2 \\ &= \sum_{\mathbf{k}} \langle a_{\mathbf{k}}^2\rangle \cos^2(\mathbf{k}\cdot\mathbf{r}) + \langle \mathrm{b}_{\mathbf{k}}^2\rangle \sin^2(\mathbf{k}\cdot\mathbf{r}) \\ &= N_k\bar{\sigma}^2,\end{aligned} \tag{S21}$$

where N_k is the number of Fourier modes. Finite sample size leads to discrete Fourier domain sampling with a step size of $\Delta k = 2\pi/\sqrt{A}$, thus within the cutoff frequency, the number of Fourier modes is $N_k = \frac{\pi k_c^2}{\Delta k^2} = \frac{k_c^2 A}{4\pi}$. The coefficient of variation of the sample is

$$R = \frac{\sqrt{\langle \Delta f(\mathbf{r})^2\rangle}}{a_0} = \frac{k_c\bar{\sigma}}{2\sqrt{\pi}}A^{\frac{3}{2}}. \tag{S22}$$

Equation (S23) then becomes

$$N_{\min}^{\mathrm{DI}} \approx \frac{k_c^2}{2\pi R^2} A \max_{|\mathbf{k}_i|<k} \frac{\beta^{\mathrm{DI}}(0)}{\left[\beta^{\mathrm{DI}}(\mathbf{k}_i)\right]^2}\gamma^2, \tag{S23}$$

where we used $a_0 = 1/A$. The right-hand side is now explicitly proportional to the sample area because R is independent of it.

Finally, we normalize the photon number to the Airy disk area $\pi\left(\frac{0.61\lambda}{\mathrm{NA}}\right)^2$, obtaining

$$n_{\min}^{\mathrm{DI}} \approx \max_{|\mathbf{k}_i|<k} \frac{8\times 0.61^2\pi^2}{R^2}\frac{\beta^{\mathrm{DI}}(0)}{\left[\beta^{\mathrm{DI}}(\mathbf{k}_i)\right]^2}\gamma^2, \tag{S24}$$

which is Eq. (8a) in the main text. For hybrid FDD, similar analysis leads to

$$n_{\min}^{\mathrm{FDD}}(\alpha, k_a) \approx \max_{|\mathbf{k}_i|<k} \frac{8\times 0.61^2\pi^2}{R^2}\left[\sum_{l=0}^{5}\frac{\left[\beta_l^H(\mathbf{k}_i)\right]^2}{\beta_l^H(0)}\right]^{-1}\gamma^2, \tag{S25}$$

which is a function of α and k_a. The overall minimum photon number should be optimized over α and k_a, thus leads to Eq. (8b) in the main text.

S3. Image reconstruction algorithm for FDD and DI dcv.

Derivation of image reconstruction algorithm

For FDD, our task is to find the coefficients $C_l(\mathbf{k})$ for linear image reconstruction $\tilde{g}^{\mathrm{FDD}}(\mathbf{k}) = \sum_{l=0}^{5} C_l(\mathbf{k})\,\tilde{g}_l(\mathbf{k})$ (Eq. (9a)), to minimize the mean square error (MSE) between $\tilde{g}^{\mathrm{FDD}}(\mathbf{k})$ and the ground truth $N\tilde{f}(\mathbf{k})$, i.e.,

$$\begin{aligned}\mathrm{MSE}(\mathbf{k}) &= \left\langle \left|N\tilde{f}(\mathbf{k}) - \tilde{g}^{\mathrm{FDD}}(\mathbf{k})\right|^2 \right\rangle \\ &= \left\langle \left|N\tilde{f}(\mathbf{k}) - \sum_l C_l(\mathbf{k})\tilde{g}_l(\mathbf{k})\right|^2 \right\rangle.\end{aligned} \tag{S26}$$

Substituting the photon density of raw FDD images $\tilde{g}_l(\mathbf{k}) = N\,\beta_l^H(\mathbf{k})\,\tilde{f}(\mathbf{k}) + \tilde{n}_l(\mathbf{k})$ into Eq. (S26), we have

$$\begin{aligned}\mathrm{MSE}(\mathbf{k}) &= \left\langle \left|N\tilde{f}(\mathbf{k}) - \sum_l C_l(\mathbf{k})\left[N\beta_l^H(\mathbf{k})\tilde{f}(\mathbf{k}) + \tilde{n}_l(\mathbf{k})\right]\right|^2 \right\rangle \\ &= N^2\left|\tilde{f}(\mathbf{k}) - \sum_l C_l(\mathbf{k})\beta_l^H(\mathbf{k})\tilde{f}(\mathbf{k})\right|^2 \\ &\quad + \sum_l \left[C_l(\mathbf{k})\right]^2\left\langle\left|\tilde{n}_l(\mathbf{k})\right|^2\right\rangle\end{aligned} \tag{S27}$$

The noise level $\tilde{n}_l(\mathbf{k})$ can be evaluated as follows. The mean photon number on the ith pixel of the detecting camera, located at $\mathbf{r}_i$, is $\langle g^{\mathrm{DI}}(\mathbf{r}_i)\rangle\Delta x^2$, while the noise photon number is $n_l(\mathbf{r}_i)\Delta x^2$. For Poisson shot noise, $\langle n_l(\mathbf{r}_i)\rangle = 0$ and $\langle n_l(\mathbf{r}_i)n_l(\mathbf{r}_{i'})\rangle = \frac{1}{\Delta x^2}\langle g_l(\mathbf{r}_i)\rangle\delta_{ii'}$. In the Fourier domain, $\tilde{n}_l(\mathbf{k}) = \iint n_l(\mathbf{r})e^{-i\mathbf{k}\cdot\mathbf{r}}d^2\mathbf{r}$, thus $\langle\tilde{n}_l(\mathbf{k})\rangle = 0$ and

$$\begin{aligned}\langle|\tilde{n}_l(\mathbf{k})|^2\rangle &= \iint\iint \langle n_l(\mathbf{r})n_l(\mathbf{r}')\rangle e^{-i\mathbf{k}\cdot(\mathbf{r}-\mathbf{r}')}d^2\mathbf{r}d^2\mathbf{r}' \\ &= \sum_{i,i'}\langle n_l(\mathbf{r}_i)n_l(\mathbf{r}_{i'})\rangle e^{-i\mathbf{k}\cdot(\mathbf{r_i}-\mathbf{r_{i'}})}\Delta x^4 \\ &= \sum_{i,i'}\langle g_l(\mathbf{r}_i)\rangle\delta_{ii'}e^{-i\mathbf{k}\cdot(\mathbf{r_i}-\mathbf{r_{i'}})}\Delta x^2 \\ &= \iint\langle g_l(\mathbf{r})\rangle d^2\mathbf{r} = N_l,\end{aligned} \tag{S28}$$

where $N_l = N\,\beta_l^H(0) \approx \tilde{g}_l(0)$ is the total photon number recorded in the lth image. Then, $\frac{\partial\mathrm{MSE}(\mathbf{k})}{\partial C_l(\mathbf{k})} = 0$ leads to

$$N^2\frac{\partial}{\partial C_l(\mathbf{k})}\left|\tilde{f}(\mathbf{k}) - \sum_m C_m(\mathbf{k})\beta_m^H(\mathbf{k})\tilde{f}(\mathbf{k})\right|^2 + 2C_l(\mathbf{k})\tilde{g}_l(0) = 0. \tag{S29}$$

Noticing that β_l is real, and assuming C_l is real,

$$\begin{aligned}&N^2\left[-2\left[\tilde{f}(\mathbf{k}) - \sum_m C_m(\mathbf{k})\beta_m^H(\mathbf{k})\tilde{f}(\mathbf{k})\right]\beta_l^H(\mathbf{k})\tilde{f}^*(\mathbf{k})\right] \\ &+ 2C_l(\mathbf{k})\tilde{g}_l(0) = 0.\end{aligned} \tag{S30}$$

Thus,

$$N^2\left|\tilde{f}(\mathbf{k})\right|^2\beta_l^H(\mathbf{k})\left[1 - \sum_m C_m(\mathbf{k})\beta_m^H(\mathbf{k})\right] - C_l(\mathbf{k})\tilde{g}_l(0) = 0. \tag{S31}$$

By rearranging the terms, we obtain a system of linear equations

$$A_l C_l(\mathbf{k}) + \sum_m \beta_m^H(\mathbf{k})C_m(\mathbf{k}) = 1, \quad (l = 0, 1, \ldots, 5) \tag{S32}$$

where $A_l = \frac{\tilde{g}_l(0)}{N^2\,|\tilde{f}(\mathbf{k})|^2\,\beta_l^H(\mathbf{k})}$. The solution of Eq. (S32) is

$$C_l(\mathbf{k}) = \frac{\beta_l^H(\mathbf{k})/\beta_l^H(0)}{\sum_{m=0}^{5}\left[\beta_m^H(\mathbf{k})\right]^2/\beta_m^H(0) + \frac{1}{N\left|\tilde{f}(\mathbf{k})\right|^2}}, \tag{S33}$$

which is consistent with Eq. (**??**) in the main text. For DI dcv, assuming $\tilde{g}^{\mathrm{DI\,dcv}}(\mathbf{k}) = C^{\mathrm{DI}}(\mathbf{k})\,\tilde{g}^{\mathrm{DI}}(\mathbf{k})$ and following the same procedure from Eq. (S26) to Eq. (S33), Eq. (10) can be derived, which is just a conventional Wiener filter (20).

The estimator derived in this sub-section is asymptotically unbiased. Substituting $\langle\tilde{g}_l(\mathbf{k})\rangle = N\,\beta_l^H(\mathbf{k})\,\tilde{f}(\mathbf{k})$ into

Eq. (9a), we see that the mean of the estimator is

$$\begin{aligned}\left\langle \tilde{g}^{\mathrm{FDD}}(\mathbf{k})\right\rangle &= \sum_{l=0}^{5} C_l(\mathbf{k})\ \langle \tilde{g}_l(\mathbf{k})\rangle \\ &= \frac{\sum_{l=0}^{5}\left[\beta_l^H(\mathbf{k})\right]^2/\beta_l^H(0)}{\sum_{m=0}^{5}\left[\beta_m^H(\mathbf{k})\right]^2/\beta_m^H(0)+\frac{1}{N\left|\tilde{f}(\mathbf{k})\right|^2}} N\tilde{f}(\mathbf{k}) \\ &= \frac{\left(\mathrm{FI}_{\mathrm{hybrid}}^{\mathrm{FDD}}\right)_{a_{\mathbf{k}},a_{\mathbf{k}}}}{\left(\mathrm{FI}_{\mathrm{hybrid}}^{\mathrm{FDD}}\right)_{a_{\mathbf{k}},a_{\mathbf{k}}}+\frac{1}{2a_0^2N\left|\tilde{f}(\mathbf{k})\right|^2}} N\tilde{f}(\mathbf{k})\end{aligned} \tag{S34}$$

Eq. (S33) and Eq. (5) are used in the last two steps. Eq. (S34) shows that the estimator is biased for low photon numbers due to the second term in the denominator. This bias scales as $1/N$. The second line of Eq. (S34) implies that the bias term plays essentially the same role as ϵ in the image reconstruction algorithm (Eq. (9c)). If the high frequency components of the sample can be successfully reconstructed, that means ϵ is relatively small, thus the bias is negligible.

Estimation accuracy saturates CRB

Here we prove that the estimator for the Fourier coefficient is asymptotically unbiased and saturates CRB of hybrid FDD, in the limit of large photon number.

Firstly, an estimator of the parameters $a_{\mathbf{k}}$ and $b_{\mathbf{k}}$ should be constructed. Note that

$$\begin{aligned}\tilde{f}(\mathbf{k})+\tilde{f}(-\mathbf{k}) &= 2\iint f(\mathbf{r})\cos(\mathbf{k}\cdot\mathbf{r})d^2\mathbf{r} \\ &= 2a_{\mathbf{k}}\iint \cos^2(\mathbf{k}\cdot\mathbf{r})d^2\mathbf{r} = \frac{a_{\mathbf{k}}}{a_0}.\end{aligned} \tag{S35}$$

In the last step, we have used $\iint \cos^2(\mathbf{k}\cdot\mathbf{r})\,d^2\mathbf{r} = \frac{A}{2} = \frac{1}{2a_0}$, which follows from the normalization condition of $f(\mathbf{r})$. Similarly, we have $i\tilde{f}(\mathbf{k}) - i\tilde{f}(-\mathbf{k}) = \frac{b_{\mathbf{k}}}{a_0}$. Since the reconstructed image $\tilde{g}^{\mathrm{FDD}}(\mathbf{k})$ is an asymptotically unbiased estimator for the ground truth $N\tilde{f}(\mathbf{k})$, in the limit of large photon number, $\epsilon_{\mathrm{FDD}}(\mathbf{k})\to 0$, an asymptotically unbiased estimator for $a_{\mathbf{k}}$ can be constructed as

$$\hat{a}_{\mathbf{k}} = \frac{a_0}{N}\left[\tilde{g}^{\mathrm{FDD}}(\mathbf{k})+\tilde{g}^{\mathrm{FDD}}(-\mathbf{k})\right]. \tag{S36}$$

It then follows from Eq. (9a) that $\hat{a}_{\mathbf{k}} = \frac{a_0}{N}\sum_l C_l\left[\tilde{g}_l(\mathbf{k})+\tilde{g}_l(-\mathbf{k})\right]$. Thus, the variance of this estimator can be calculated as

$$\left\langle \Delta a_{\mathbf{k}}^2\right\rangle = \frac{a_0^2}{N^2}\sum_l C_l^2\left[\langle|\tilde{n}_l(\mathbf{k})|^2\rangle+\langle|\tilde{n}_l(-\mathbf{k})|^2\rangle\right] \tag{S37}$$

where the noise term $\langle|\tilde{n}_l(\pm\mathbf{k})|^2\rangle = N\,\beta_l^H(0)$ is derived in Eq. (S28). Substituting the expression of C_l in Eq. (S33) here, we find that,

$$\begin{aligned}\left\langle \Delta a_{\mathbf{k}}^2\right\rangle &= \frac{2a_0^2}{N}\frac{\sum_{l=0}^{5}\left[\beta_l^H(\mathbf{k})\right]^2/\beta_l^H(0)}{\left[\sum_{m=0}^{5}\left[\beta_m^H(\mathbf{k})\right]^2/\beta_m^H(0)+\frac{1}{N\left|\tilde{f}(\mathbf{k})\right|^2}\right]^2} \\ &= \frac{1}{N}\frac{\left(\mathrm{FI}_{\mathrm{hybrid}}^{\mathrm{FDD}}\right)_{a_{\mathbf{k}},a_{\mathbf{k}}}}{\left[\left(\mathrm{FI}_{\mathrm{hybrid}}^{\mathrm{FDD}}\right)_{a_{\mathbf{k}},a_{\mathbf{k}}}+\frac{1}{2a_0^2N\left|\tilde{f}(\mathbf{k})\right|^2}\right]^2}\end{aligned} \tag{S38}$$

Comparing with Eqs. (5)-(7) , we see that $\left\langle \Delta a_{\mathbf{k}}^2\right\rangle = \frac{1}{N}\left(\mathrm{CRB}_{\mathrm{hybrid}}^{\mathrm{FDD}}\right)_{a_{\mathbf{k}},a_{\mathbf{k}}}$ in the limit of large photon number N, i.e., this estimator saturates CRB. At low photon numbers, however, the estimator becomes biased (Eq. (S34)). In this regime, Eq. (S38) shows that the variance is reduced at the expense of increased bias. Similar analysis works for $b_{\mathbf{k}}$ as well.

REFERENCES

1. M. Born, E. Wolf, *Principles of Optics: Electromagnetic Theory of Propagation, Interference and Diffraction of Light*, (Cambridge Univ. Press, ed. 7, 2013).

2. J. W. Goodman, *Introduction to Fourier Optics*, (McGraw-Hill, ed. 2, 1996).

3. W. Zhao, S. Zhao, L. Li, X. Huang, S. Xing, Y. Zhang, G. Qiu, Z. Han, Y. Shang, D. Sun, C. Shan, R. Wu, L. Gu, S. Zhang, R. Chen, J. Xiao, Y. Mo, J. Wang, W. Ji, X. Chen, B. Ding, Y. Liu, H. Mao, B.-L. Song, J. Tan, J. Liu, H. Li, L. Chen, Sparse deconvolution improves the resolution of live-cell super-resolution fluorescence microscopy. *Nat. Biotechnol.* **40**, 606–617 (2022).

4. M. Tsang, R. Nair, X.-M. Lu, Quantum theory of superresolution for two incoherent optical point sources. *Phys. Rev. X* **6**, 031033 (2016).

5. C. W. Helstrom, *Quantum Detection and Estimation Theory* (Academic Press, 1976).

6. J. Řehaček, Z. Hradil, B. Stoklasa, M. Paúr, J. Grover, A. Krzic, L. L. Sánchez-Soto, Multiparameter quantum metrology of incoherent point sources: Towards realistic superresolution. *Phys. Rev. A* **96**, 062107 (2017).

7. F. Yang, R. Nair, M. Tsang, C. Simon, A. I. Lvovsky, Fisher information for far-field linear optical superresolution via homodyne or heterodyne detection in a higher-order local oscillator mode. *Phys. Rev. A* **96**, 063829 (2017).

8. C. Lupo, S. Pirandola, Ultimate precision bound of quantum and subwavelength imaging. *Phys. Rev. Lett.* **117**, 190802 (2016).

9. I. Ozer, M. R. Grace, P.-A. Blanche, S. Guha, Adaptive super-resolution imaging without prior knowledge using a programmable spatial-mode sorter. (2024).

10. M. Paúr, B. Stoklasa, Z. Hradil, L. L. Sánchez-Soto, J. Rehacek, Achieving the ultimate optical resolution. *Optica* **3**, 1144–1147 (2016).

11. L. Santamaria, F. Sgobba, C. Lupo, Single-photon sub-rayleigh precision measurements of a pair of incoherent sources of unequal intensity. *Op. Quantum* **2**, 46–56 (2024).

12. C. Rouvière, D. Barral, A. Grateau, I. Karuseichyk, G. Sorelli, M. Walschaers, N. Treps. Ultra-sensitive separation estimation of optical sources, *Optica* **11**, 166–170 (2024).

13. N. Deshler, I. Ozer, A. Ashok, S. Guha. Experimental demonstration of a quantum-optimal coronagraph using spatial mode sorters. *Optica* **12**, 518–529 (2025).

14. F. Yang, A. Tashchilina, E. S. Moiseev, C. Simon, A. I. Lvovsky, Far-field linear optical superresolution via heterodyne detection in a higher-order local oscillator mode. *Optica* **3**, 1148–1152 (2016).

15. M. Tsang, Subdiffraction incoherent optical imaging via spatial-mode demultiplexing. *New J. Phys.* **19**, 023054 (2017).

16. A. A Pushkina, G. Maltese, J. I Costa-Filho, P. Patel, A. I Lvovsky, Superresolution linear optical imaging in the far field. *Phys. Rev. Lett.* **127**, 253602 (2021).

17. J. Frank, A. Duplinskiy, K. Bearne, A. I. Lvovsky, Passive superresolution imaging of incoherent objects. *Optica* **10**, 1147–1152 (2023).

18. A. Duplinskiy, J. Frank, K. Bearne, A. I. Lvovsky, Tsang's resolution enhancement method for imaging with focused illumination. *Light Sci. Appl.* **14**, 159 (2025).

19. M. Tsang, F. Albarelli, A. Datta, Quantum semiparametric estimation. *Phys. Rev. X* **10**, 031023 (2020).

20. J. Dong, S. Roth, B. Schiele. Deep wiener deconvolution: Wiener meets deep learning for image deblurring. *Adv. Neural Inf. Proces. Syst.* **33**, 1048–1059 (2020).

21. B. Richards, E. Wolf, Electromagnetic diffraction in optical systems, ii. structure of the image field in an aplanatic system. *Proc. R. Soc. Lond. A Math. Phys. Sci.* **253**, 358–379 (1959).

22. P. Philippe Laissue, R. A. Alghamdi, P. Tomancak, E. G. Reynaud, H. Shroff, Assessing phototoxicity in live fluorescence imaging. *Nat. Methods* **14**, 657–661 (2017).

23. W. Denk, J. H. Strickler, W. W. Webb, Two-photon laser scanning fluorescence microscopy. *Science* **248**, 73–76 (1990).

24. L. Gong, Y. Dou, S. Lin, T. Osipowicz, Z. Huang, Fourier ptychographic coherent anti-stokes raman scattering microscopy with point-scanning for super-resolution imaging. *Small Methods* **10**, e2400765 (2026).

25. M. I. Kolobov, C. Fabre, Quantum limits on optical resolution. *Phys. Rev. Lett.* **85**, 3789–3792 (2000).

26. R. Nair, M. Tsang, Far-field superresolution of thermal electromagnetic sources at the quantum limit. *Phys. Rev. Lett.* **117**, 190801 (2016).

27. O. Schwartz, J. M. Levitt, R. Tenne, S. Itzhakov, Z. Deutsch, D. Oron, Superresolution microscopy with quantum emitters. *Nano Lett.* **13**, 5832–5836 (2013).

28. R. Tenne, U. Rossman, B. Rephael, Y. Israel, A. Krupinski-Ptaszek, R. Lapkiewicz, Y. Silberberg, D. Oron, Super-resolution enhancement by quantum image scanning microscopy. *Nat. Photonics* **13**, 116–122 (2019).

29. J. Rubio, J. Dunningham, Bayesian multiparameter quantum metrology with limited data. *Phys. Rev. A* **101**, 032114 (2020).

30. J. R. Hervas, A. Z. Goldberg, A. S. Sanz, Z. Hradil, J. Řeháček, L. L. Sánchez-Soto, Beyond the quantum cramér-rao bound. *Phys. Rev. Lett.* **134**, 010804 (2025).

31. J. R. Hervas, A. Z. Goldberg, A. S. Sanz, Z. Hradil, J. Řeháček, L. L. Sánchez-Soto, Higher-order corrections to the quantum cramér-rao bound. *Phys. Rev. A* **112**, 022426 (2025).

32. J. J. Meyer, S. Khatri, D. S. França, J. Eisert, P. Faist, Quantum metrology in the finite-sample regime. *PRX Quantum* **6**, 030336 (2025).

33. J. Suzuki, Information geometrical characterization of quantum statistical models in quantum estimation theory. *Entropy* **21**, 703 (2019).

34. F. Albarelli, J. F. Friel, A. Datta, Evaluating the holevo cramér-rao bound for multiparameter quantum metrology. *Phys. Rev. Lett.* **123**, 200503, (2019).

35. L. O. Conlon, J. Suzuki, P. K. Lam, S. M. Assad, Efficient computation of the nagaoka–hayashi bound for multiparameter estimation with separable measurements. *npj Quantum Inf.* **7**, 110 (2021).

36. J. Liu, H. Yuan, X.-M. Lu, X. Wang. Quantum fisher information matrix and multiparameter estimation. *J. Phys. A Math. Theor.* **53**, 023001 (2020).
